# Prevalence and incidence of iron deficiency in European community-dwelling older adults: an observational analysis of the DO-HEALTH trial

**DOI:** 10.1007/s40520-022-02093-0

**Published:** 2022-03-18

**Authors:** Alenka Stahl-Gugger, Caroline de Godoi Rezende Costa Molino, Maud Wieczorek, Patricia O. Chocano-Bedoya, Lauren A. Abderhalden, Dominik J. Schaer, Donat R. Spahn, E. John Orav, Bruno Vellas, José A. P. da Silva, Reto W. Kressig, Andreas Egli, Heike A. Bischoff-Ferrari, for the DO-HEALTH Research Group, Heike A. Bischoff-Ferrari, Andreas Egli, Bruno Vellas, Sophie Guyonnet, René Rizzoli, Emmanuel Biver, Fanny Merminod, Reto W. Kressig, Stephanie Bridenbaugh, Norbert Suhm, José A. P. Silva, Cátia C. M. Duarte, Dieter Felsenberg, Hendrikje Börst, Gabriele Armbrecht, Michael Blauth, Anna Spicher, David T. Felson, John A. Kanis, Eugene V. Mccloskey, Elena Johansson, Bernhard Watzl, Lorenz Hofbauer, Elena Tsourdi, Martina Rauner, Uwe Siebert, John A. Kanis, Stephen M. Ferrari, Benno Gut, Marième Ba, Stéphane Etheve, Manfred Eggersdorfer, Monika Reuschling, Endel J. Orav, Walter C. Willett, JoAnn E. Manson, Bess Dawson-Hughes, Hannes B. Staehelin, Paul W. Walter, Walter Dick, Michael Fried, Arnold Eckardstein, Robert Theiler, Hans-Peter Simmen, Wolfgang Langhans, Annelies Zinkernagel, Nicolas Mueller, Oliver Distler, Klaus Graetz, Ina Nitschke, Thomas Dietrich, Walter Baer, Klara Landau, Frank Ruschitzka, Markus Manz, Peter Burckhardt

**Affiliations:** 1 Center on Aging and Mobility, University Hospital Zurich, Zurich City Hospital-Waid and University of Zurich, Zurich, Switzerland; 2 Institute of Primary Health Care (BIHAM), University of Bern, Bern, Switzerland; 3 Population Health Lab, University of Fribourg, Fribourg, Switzerland; 4 Clinic for Internal Medicine, University Hospital Zurich, Zurich, Switzerland; 5 Institute of Anesthesiology, University Hospital Zurich and University of Zurich, Zurich, Switzerland; 6 Department of Biostatistics, Harvard School of Public Health, Boston, USA; 7 Gérontopôle de Toulouse, Institut du Vieillissement, Center Hospitalo-Universitaire de Toulouse, Toulouse, France; 8 UMR INSERM 1027, University of Toulouse III, Toulouse, France; 9 Institute for Clinical and Biomedical Research (iCBR), Faculty of Medicine, University of Coimbra, Coimbra, Portugal; 10 Centro Hospitalar e Universitário de Coimbra, Coimbra, Portugal; 11 University Department of Geriatric Medicine FELIX PLATTER, and University of Basel, Basel, Switzerland; 12 Present Address: Department of Aging Medicine and Aging Research, University Hospital Zurich and University of Zurich, Raemistrasse 101, 8091 Zurich, Switzerland; 13 University Clinic for Aging Medicine, Zurich City Hospital-Waid, Zurich, Switzerland

**Keywords:** Iron deficiency, Prevalence, Incidence, Community-dwelling older adults, Europe

## Abstract

**Background and aim:**

Iron deficiency is associated with increased morbidity and mortality in older adults. However, data on its prevalence and incidence among older adults is limited. The aim of this study was to investigate the prevalence and incidence of iron deficiency in European community-dwelling older adults aged ≥ 70 years.

**Methods:**

Secondary analysis of the DO-HEALTH trial, a 3-year clinical trial including 2157 community-dwelling adults aged ≥ 70 years from Austria, France, Germany, Portugal and Switzerland. Iron deficiency was defined as soluble transferrin receptor (sTfR) > 28.1 nmol/L. Prevalence and incidence rate (IR) of iron deficiency per 100 person-years were examined overall and stratified by sex, age group, and country. Sensitivity analysis for three commonly used definitions of iron deficiency (ferritin < 45 μg/L, ferritin < 30 μg/L, and sTfR–ferritin index > 1.5) were also performed.

**Results:**

Out of 2157 participants, 2141 had sTfR measured at baseline (mean age 74.9 years; 61.5% women). The prevalence of iron deficiency at baseline was 26.8%, and did not differ by sex, but by age (35.6% in age group ≥ 80, 29.3% in age group 75–79, 23.2% in age group 70–74); *P* < 0.0001*)* and country (*P* = 0.02), with the highest prevalence in Portugal (34.5%) and the lowest in France (24.4%). As for the other definitions of iron deficiency, the prevalence ranged from 4.2% for ferritin < 30 µg/L to 35.3% for sTfR–ferritin index > 1.5. Occurrences of iron deficiency were observed with IR per 100 person-years of 9.2 (95% CI 8.3–10.1) and did not significantly differ by sex or age group. The highest IR per 100 person-years was observed in Austria (20.8, 95% CI 16.1–26.9), the lowest in Germany (6.1, 95% CI 4.7–8.0). Regarding the other definitions of iron deficiency, the IR per 100 person-years was 4.5 (95% CI 4.0–4.9) for ferritin < 45 µg/L, 2.4 (95% CI 2.2–2.7) for ferritin < 30 µg/L, and 12.2 (95% CI 11.0–13.5) for sTfR–ferritin index > 1.5.

**Conclusions:**

Iron deficiency is frequent among relatively healthy European older adults, with people aged ≥ 80 years and residence in Austria and Portugal associated with the highest risk.

**Supplementary Information:**

The online version contains supplementary material available at 10.1007/s40520-022-02093-0.

## Introduction

Iron deficiency is the most common nutrient deficiency worldwide, thus constituting a major health problem [1]. Notably, 1.6 billion people, corresponding to a fifth of the world population, are iron deficient, of which about 1 billion have a severe form with subsequent anemia [2]. While iron deficiency is a major public health problem in developing countries [3], it also affects high-income countries [4–6]. In Europe, the prevalence in adults (age 16–50 years) has been found to range from 4 to 33% [7]. A special risk group for iron deficiency are older adults, where a peak of iron deficiency has been described [8, 9].

Iron deficiency has several adverse effects in the human body, as iron plays a pivotal role in oxygen transportation to tissues and in removal of carbon dioxide [10]. Iron deficiency is suggested to be the most important contributor to nutrient deficiency anemia at older age [4]. Previous studies among older adults suggest that reduced iron store, independent of anemia status, may be associated with inflammation [11], an increased risk of physical and cognitive impairment [12, 13] and an increased overall morbidity and mortality [14–17]. In fact, all-cause mortality among severely iron deficient subjects (aged ≥ 65 years, living in long-term care facilities) is estimated to be almost twice as high as in iron sufficient persons of the same age and living conditions [15].

Regarding reliable prevalence estimates of iron deficiency among older adults, it is important to note that there is no consensus about the best marker to diagnose iron deficiency in this growing segment of the population [8, 18–20]. To address this bottle-neck, the present study assessed the prevalence and incidence of iron deficiency in a large European cohort of community-dwelling older adults using the four most common definitions of iron deficiency.

## Methods

### Participants and study design

This study is a secondary analysis of data collected as part of the DO-HEALTH clinical trial (NCT01745263). DO-HEALTH is a multicenter, double-blind, randomized controlled trial (RCT) designed to support healthy aging in European seniors. The trial examined the individual and combined effects of omega-3 fatty acids, vitamin D, and a simple home exercise program over 3 years of follow-up. A total of 2157 community-dwelling older adults were recruited in five European countries (Austria, France, Germany, Portugal, and Switzerland) from seven university centers (Basel, Berlin, Coimbra, Geneva, Innsbruck, Toulouse, and Zurich).

Briefly, DO-HEALTH included relatively healthy adults aged 70 years or older, with Mini Mental State Examination score (MMSE) ≥ 24 and sufficiently mobile to come to the study center. Key exclusion criteria for the present study were: severe liver disease (such as chronic active hepatitis B, cirrhosis of the liver or sclerosing cholangitis), severe renal impairment (creatinine clearance ≤ 15 mL/min) or dialysis, history of cancer (except non-melanoma skin cancer), or history of a cardiovascular event in the last 5 years. Details on the trial are published elsewhere [21].

### Assessments and blood samples

DO-HEALTH participants were followed for 3 years with four clinical visits (at baseline, after 1, 2, and 3 years) and phone calls every 3 months. The following variables were assessed at baseline: sex, age, years of education, smoking status, the intake of medications or supplements, body mass index (BMI), and comorbidities with the Self-Administered Comorbidity Questionnaire (SCQ, range: 0–13) [22].

Baseline and follow-up blood samples at every visit (at 1, 2, and 3 years) were collected in all participants to measure soluble transferrin receptor (sTfR), and ferritin levels. Hemoglobin level for the diagnosis of anemia was only assessed at baseline. Anemia was defined as hemoglobin < 130 g/L for men and < 120 g/L for women, according to World Health Organization (WHO) guidelines [23].

The sTfR measurement was done by particle-enhanced immunoturbidimetric assay, with coefficient of variation of 2.2% at 2.6 mg/L and 1.8% at 7.1 mg/L. Ferritin concentrations were measured with Elecsys Ferritin Test on a cobas e 801 analyser using ElektroChemiLumineszenz-ImmunoAssay “ECLIA” technology, with coefficients of variation of 3.5% at 154 µg/L and 4.5% at 947 µg/L. All blood samples were analyzed centrally in the same laboratory.

### Definition of iron deficiency and outcomes

Primary outcomes were the baseline prevalence and incidence rate of iron deficiency. Iron deficiency was assessed every year over the 3-year follow-up, so that patients could have anywhere from 0 to 3 occurrences. For the main definition of iron deficiency, we used sTfR > 28.1 nmol/L, which, unlike ferritin, is not influenced by chronic diseases or age [24–26]. In addition, we performed sensitivity analysis for three commonly used definitions of iron deficiency: ferritin < 45 μg/L [8, 18, 27, 28], ferritin < 30 μg/L [19, 29, 30], and sTfR–ferritin index (calculated as sTfR/log ferritin) > 1.5 [31].

### Statistical analysis

Prevalence (absolute numbers and proportions) of iron deficiency at baseline and the incidence of iron deficiency were estimated and compared by sex, age group (70–74, 75–59, and ≥ 80 years), and country (Austria, France, Germany, Portugal, and Switzerland). The incidence rate (IR) of iron deficiency was based on only subjects with iron sufficiency at baseline. It was calculated as the total number of years during which iron deficiency was observed to occur, divided by the total number of years that subjects were followed. A subject who was followed for 3 years could contribute up to 3 occurrences of iron deficiency. Analyzes were stratified by age group and sex, as prior studies found that the prevalence of iron deficiency increases with age [32] and is more frequent in women [5, 17]. We also investigated the prevalence and incidence of iron deficiency by country as diet and potentially iron intake are culturally diverse [21]. Prevalence of iron deficiency was compared between sex, age group, and country using a Chi-square test. IRs and 95% confidence intervals (CI) over the study period were estimated using over-dispersed Poisson regression models. An offset of the logarithm of each participant’s time (years) in the study was included in the models. IR and 95% CI were also estimated for each subgroup (sex, age group, and country) using over-dispersed Poisson regression models. Statistical significance was set at *P* value of < 0.05, and reported *P* values are two sided. Statistical analysis was performed using SAS v9.4.

## Results

### Baseline characteristics

Out of 2157 DO-HEALTH participants, 2141 had sTfR and ferritin measured at baseline and were included in the study. Table 1 gives an overview of the baseline characteristics: 61.5% of the participants were women and the mean age was 74.9 years (SD ± 4.5). Overall, mean BMI was 26.3 kg/m^2^ (SD ± 4.3), mean serum hemoglobin was 139.8 g/L (SD ± 12.4), and mean number of comorbidities was 1.7 (SD ± 1.4). A minority of participants were current smokers (5.8%, 125/2141), took iron supplements (5.6%, 120/2141), or had anemia (6.5%, 140/2141).

**Table 1:** Baseline characteristics

	Overall
*N*	2141
Women, *N* (%)	1317 (61.5)
Men, *N* (%)	824 (38.5)
Age, years, mean (SD)	74.9 (4.5)
Years of education, mean (SD)	12.7 (4.3)
Current smokers, *N* (%)	125 (5.8)
Iron supplement, *N* (%)	120 (5.6)
BMI, kg/m^2^, mean (SD)	26.3 (4.3)
Hb, g/L, mean (SD)	139.8 (12.4)
Anemia^a^, *N* (%)	140 (6.5)
Number of comorbidities^b^, mean (SD)	1.7 (1.4)
Self-reported chronic diseases^b^, *N* (%)	
Osteoarthritis	944 (44.1)
High blood pressure	834 (39.0)
Back pain	762 (35.6)
Heart disease	258 (12.1)
Depression	175 (8.2)
Ulcer or stomach disease	160 (7.5)
Diabetes	149 (7.0)
Rheumatoid arthritis	117 (5.5)
Lung disease	107 (5.0)
Kidney disease	54 (2.5)
Liver disease	35 (1.6)
Cancer	26 (1.2)

*BMI* body mass index, *Hb* hemoglobin, *N* number, *SD* standard deviation

^a^Definition of anemia: < 130 g/L for men, < 120 g/L for women

^b^Comorbidities were assessed by the Self-Administered Comorbidity Questionnaire (SCQ) [22]

### Prevalence of iron deficiency at baseline

The prevalence of iron deficiency at baseline—according to the four definitions—is illustrated in Table 2: 26.8% (573/2141) of the participants had sTfR > 28.1 nmol/L, 9.3% (199/2141) had ferritin < 45 µg/L, 4.2% (90/2141) had ferritin < 30 µg/L, and 35.3% (755/2141) had sTfR–ferritin index > 1.5. Compared to men, women had a nearly three times higher prevalence of ferritin < 45 µg/L than men (12.2% vs. 4.6%; *P* < 0.0001) and a significantly higher prevalence of sTfR–ferritin index > 1.5 (40.3% vs. 27.2%; *P* < 0.0001). The oldest age group (80 +) had a significantly higher prevalence of iron deficiency, except by the definition ferritin < 30 µg/L.

**Table 2:** Prevalence of iron deficiency at baseline for the overall sample, by sex and age group

	Overall	Sex		*P*^a^	Age group			*P*^a^
		Men	Women		70–74	75–79	80 +	
*N*	2141	824	1317		1227	588	326	
sTfR > 28.1 nmol/L, *N* (%)	573 (26.8)	210 (25.5)	363 (27.6)	0.29	285 (23.2)	172 (29.3)	116 (35.6)	< 0.0001
Ferritin < 45 µg/L, *N* (%)	199 (9.3)	38 (4.6)	161 (12.2)	< 0.0001	109 (8.9)	44 (7.5)	46 (14.1)	0.003
Ferritin < 30 µg/L, *N* (%)	90 (4.2)	21 (2.6)	69 (5.2)	0.003	49 (4.0)	20 (3.4)	21 (6.4)	0.08
sTfR–ferritin index^b^ > 1.5, *N* (%)	755 (35.3)	224 (27.2)	531 (40.3)	< 0.0001	402 (32.8)	210 (35.7)	143 (43.9)	0.0009

*N* number, *P* *P* value, *sTfR* soluble transferrin receptor

^a^Differences between groups were assessed using a *χ*^2^ test

^b^Definition of sTfR–ferritin index: sTfR/log ferritin

### Prevalence of iron deficiency anemia at baseline

**Fig. 1:**
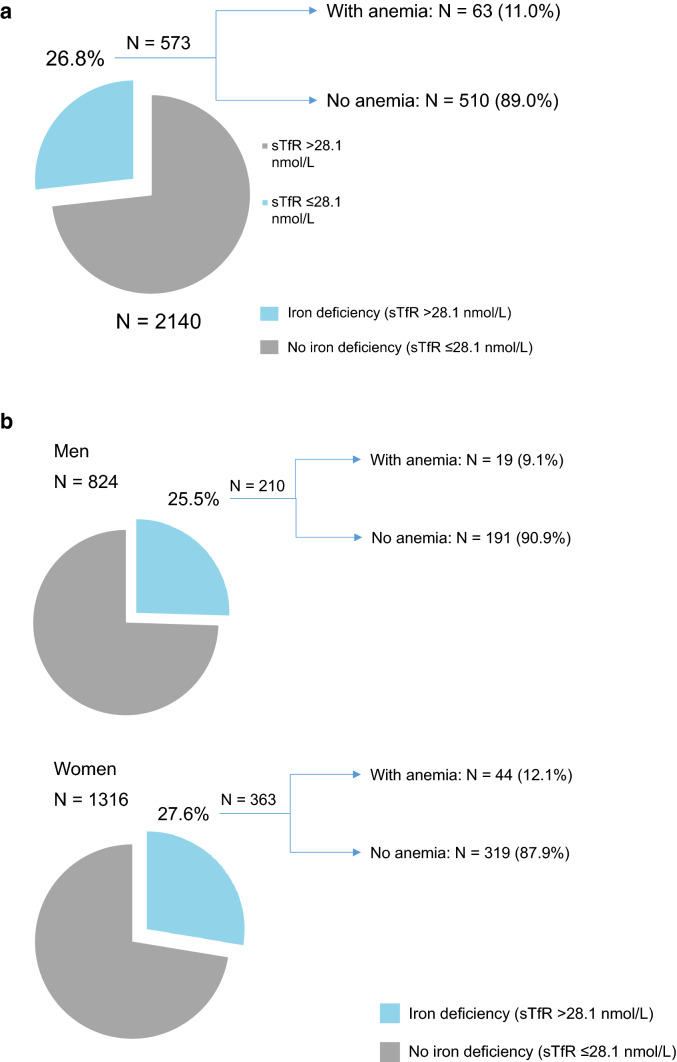
Prevalence of iron deficiency (sTfR > 28.1 nmol/L) with or without anemia at baseline, overall (**A**) and by sex (**B**). There is one missing hemoglobin level among women without iron deficiency. Therefore, the sample size is 2140

Among the iron deficient population (sTfR > 28.1 nmol/L), we found that 11.0% (63/573) of them had anemia (Fig. 1A). Among iron deficient men, only 9.1% (19/210) had anemia. Among iron deficient women, 12.1% (44/363) had also anemia (Fig. 1B).

### Prevalence of iron deficiency by country

**Fig. 2:**
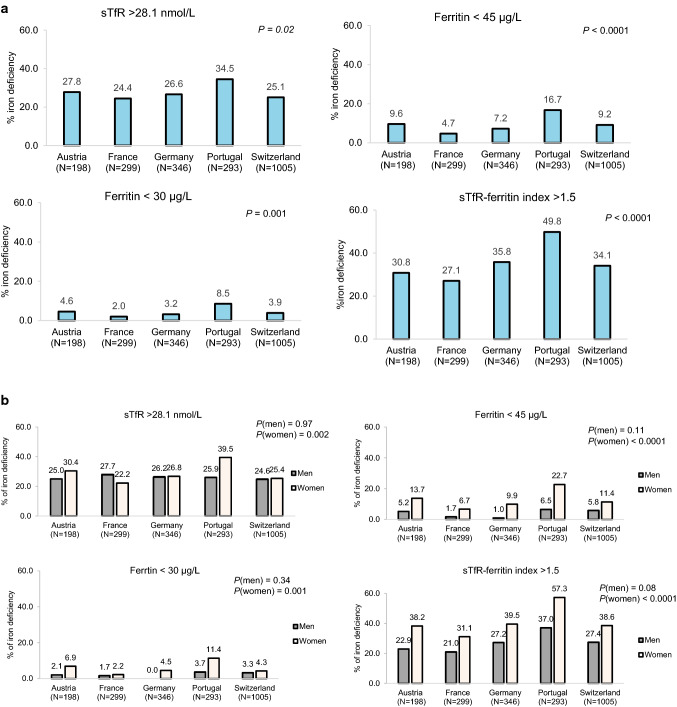
Prevalence (%) of iron deficiency (sTfR > 28.1 nmol/L, ferritin < 45 µg/L, ferritin < 30 µg/L, and sTfR–ferritin index > 1.5) by country (**A**) and by country and sex at baseline (**B**). *P* values are from Chi-square test

The baseline prevalence of iron deficiency differed significantly between countries for all four definitions. The highest prevalence was in Portugal (range: 8.5% for ferritin < 30 µg/L to 49.8% for sTfR–ferritin index > 1.5), and the lowest in France (range: 2.0% for ferritin < 30 µg/L to 27.1% for sTfR–ferritin index > 1.5), see Fig. 2A**.**

Figure 2B illustrates the prevalence of iron deficiency stratified by country and sex. Among women, Portugal had the highest prevalence of iron deficiency for the four definitions (range: 11.4% for ferritin < 30 µg/L to 57.3% for sTfR–ferritin index > 1.5). Among men, no significant difference in the prevalence of iron deficiency by country was observed.

### Incidence of iron deficiency over the study period

Over the 3 years of follow-up, there were 390 occurrences of iron deficiency (defined by sTfR > 28.1 nmol/L) among participants with iron sufficiency at baseline. This made an overall IR per 100 person-years of 9.2 (95% CI 8.3–10.1). For ferritin < 45 µg/L, there were 234 occurrences with an overall IR of 4.5 (95% CI 4.0–4.9). For ferritin < 30 µg/L, there were 134 occurrences with an overall IR of 2.4 (95% CI 2.2–2.7). There were 452 occurrences using sTfR–ferritin index > 1.5 with an IRs per 100 person-years of 12.2 (95% CI 11.0–13.5) (Table 3).

**Table 3:** Incidence rate of iron deficiency over the study period among participants with iron sufficiency at baseline

	sTfR > 28.1 nmol/L				Ferritin < 45 µg/L				Ferritin < 30 µg/L				sTfR–ferritin index > 1.5			
	*N* participants with iron sufficiency at BL	Occurrences of ID	IR (95% CI) per 100 person-years	*P*	*N* participants with iron sufficiency at BL	Occurrences of ID	IR (95% CI) per 100 person-years	*P*	*N* participants with iron sufficiency at BL	Occurrences of ID	IR (95% CI) per 100 person-years	*P*	*N* participants with iron sufficiency at BL	Occurrences of ID	IR (95% CI) per 100 person-years	*P*
**Overall**	1568	390	9.2 (8.3–10.1)	–	1942	234	4.5 (4.0–4.9)	–	2051	134	2.4 (2.2–2.7)	–	1386	452	12.2 (11.0–13.5)	–
Sex																
Men	614	158	9.5 (8.1–11.1)	Ref	786	64	3.0 (2.5–3.6)	Ref	803	41	1.9 (1.6–2.3)	Ref	600	169	10.5 (8.9–12.4)	Ref
Women	954	232	9.0 (7.9–10.2)	0.63	1156	170	5.5 (4.8–6.2)	** < 0.0001**	1248	93	2.8 (2.4–3.2)	**0.002**	786	283	13.5 (11.8–15.4)	**0.02**
Age group																
70–74	942	225	8.8 (7.7–10.0)	Ref	1118	126	4.1 (3.6–4.8)	Ref	1178	66	2.1 (1.8–2.4)	Ref	825	240	10.8 (9.4–12.4)	Ref
75–79	416	104	9.2 (7.6–11.1)	0.70	544	55	3.7 (3.0–4.6)	0.42	568	33	2.1 (1.7–2.7)	0.80	378	131	13.0 (10.8–15.8)	0.12
80 +	210	61	11.1 (8.6–14.3)	0.10	280	53	7.3 (5.6–9.4)	** < 0.0001**	305	35	4.4 (3.4–5.8)	** < 0.0001**	183	81	17.2 (13.2–22.5)	**0.001**
Country																
Austria	143	79	20.8 (16.1–26.9)	** < 0.0001**	179	18	3.9 (2.7–5.6)	0.36	189	10	2.1 (1.4–3.0)	0.85	137	100	28.0 (21.6–36.4)	** < 0.0001**
France	226	39	6.7 (5.0–8.9)	0.46	285	16	2.2 (1.6–3.0)	**0.0003**	293	9	1.2 (0.9–1.7)	**0.01**	218	51	9.1 (6.9–12.0)	0.46
Germany	254	42	6.1 (4.7–8.0)	0.20	321	32	3.7 (2.8–4.8)	0.12	335	19	2.1 (1.6–2.8)	0.88	222	47	7.9 (5.9–10.5)	0.13
Portugal	192	72	13.9 (10.8–17.8)	** < 0.0001**	244	50	7.5 (5.8–9.7)	**0.0006**	268	39	5.4 (4.1–7.1)	** < 0.0001**	147	69	17.5 (13.0–23.4)	**0.0006**
Switzerland	753	158	7.6 (6.6–8.8)	Ref	913	118	4.7 (4.0–5.5)	Ref	966	57	2.2 (1.8–2.5)	Ref	662	185	10.3 (8.9–12.0)	Ref

Significant *P*-values (*P* < 0.05) are highlighted in bold

*CI* confidence interval, *ID* iron deficiency, *IR* incidence rate, *N* absolute number, *P* *P* value, *Ref* reference group, *sTfR* soluble transferrin receptor

Rates and *P* values from over-dispersed Poisson regression models with incident iron deficiency across 3 years as outcome and with each participant’s time in the study as an exposure offset

In three out of the four iron deficiency definitions, women had a significantly higher IR compared to men: ferritin < 45 µg/L (IR 5.5 [95% CI 4.8–6.2] vs. 3.0 [95% CI 2.5–3.6], *P* < 0.0001), ferritin < 30 µg/L (IR 2.8 [95% CI 2.4–3.2] vs. 1.9 [95% CI 1.6–2.3], *P* = 0.002) and sTfR–ferritin index > 1.5 (IR 13.5 [95% CI 11.8–15.4] vs. 10.5 [95% CI 8.9–12.4], *P* = 0.02). The 80 + age group had a significantly higher IR when compared to the age group 70–74 years in the same three out of four definitions (Table 3).

### Incidence of iron deficiency by country

For the comparison analysis between different countries, Switzerland was the reference group as this country had the highest number of participants (*N* = 1005). The highest IR of sTfR > 28.1 nmol/L was found in Austria with 20.8 (95% CI 16.1–26.9) and the lowest in Germany with 6.1 (95% CI 4.7–8.0). The same pattern was found for sTfR–ferritin index. The highest IR of ferritin < 45 µg/L was observed in Portugal with 7.5 (95%CI 5.8–9.7) and the lowest in France with 2.2 (95% CI 1.6–3.0). Similar pattern was found for ferritin < 30 µg/L (Table 3).

## Discussion

This study examined the prevalence and incidence of iron deficiency among relatively healthy adults aged 70 years and older recruited in five European countries for the DO-HEALTH trial. To date, this is the first study to report incidence rates of iron deficiency (with or without anemia) among community-dwelling older adults in Europe. Depending on its definition, we found an overall prevalence of iron deficiency ranging from 4.2% (ferritin < 30 µg/L) to 35.3% (sTfR–ferritin index > 1.5). The prevalence of iron deficiency was higher among women and the oldest age group (80 +) for all definitions. In addition, the prevalence of iron deficiency was the highest in Portugal and the lowest in France for the four definitions. Notably, at baseline, the vast majority of older adults with prevalent iron deficiency (sTfR > 28.1 nmol/L) did not present with concomitant anemia (89.0% of iron deficient participants were non-anemic, 87.9% among women, and 90.9% among men). The IR per 100 person-years varied from 2.4 (95% CI 2.2–2.7) to 12.2 (95% CI 11.0–13.5), depending on the definition of iron deficiency. For the definition sTfR > 28.1 nmol/L, the IRs of iron deficiency were not statistically different by sex or age group. However, the incidence of iron deficiency was significantly higher in women compared to men and in the oldest age group (80 + years) compared to the youngest group (70–74 years) when taking the other three definitions of iron deficiency (ferritin < 45 µg/L, ferritin < 30 µg/L, and sTfR–ferritin index > 1.5).

Our study adds to the existing evidence by providing data on the prevalence of iron deficiency among community-dwelling older adults. For instance, most prior European studies on iron status focused on children, premenopausal or pregnant women [33], or young adults [34, 35]. To compare our results, a recently published observational study among older adults aged > 50 years from England found a prevalence of non-anemic iron deficiency (ferritin < 30 µg/L) of 10.9% in women and 6.3% in men [17]. The population-based EMPIRE study among community-dwelling adults from Portugal found a prevalence of iron deficiency (ferritin < 30 μg/L) of 30.2% among adults aged 65–79 years (*N* = 1187) and of 42.8% among adults aged ≥ 80 years (*N* = 430) [36]. These estimates are considerably higher compared with our results, which may be best explained by the selection of relatively healthy older adults for the DO-HEALTH trial, which is confirmed by the lower prevalence of comorbidities than in the EMPIRE population (e.g., 18% of the EMPIRE population aged 65–79 years reported history of diabetes, 34% history of kidney failure, and 20% history of lung diseases; while in DO-HEALTH, the prevalence of diabetes was 7%, kidney disease was 2.5%, and lung diseases was 5%).

Our findings revealed differences in the prevalence and incidence of iron deficiency among relatively healthy older adults between the five European countries (Austria, France, Germany, Portugal, and Switzerland). Based on sTfR (> 28.1 nmol/L), older adults from France had the lowest prevalence of iron deficiency with 24.4%, and Portugal had the highest prevalence with 34.5%. Our results about the iron-deficiency prevalence by country should be interpreted carefully. Notably, we could not find population-based data from other cohorts enrolling older European adults, to establish if our by-country differences among relatively healthy older adults are maintained at the population level. Participants in DO-HEALTH were not selected from a random sample of the population of each country; therefore, our results might not be representative of the population at large. Differences in factors affecting self-selection may contribute to the intercountry differences. In addition, regarding the difference in iron deficiency by country as a possible indicator of health disparities between countries, there is some support of an association between iron deficiency and low socioeconomic status [37–39]. Even though this evidence is based on children and young women, this could be one of the factors contributing to the high prevalence and incidence of iron deficiency in Portugal. However, participants from Austria, considered a country with a higher socio-economic status, had a high incidence of iron-deficiency. As dietary patterns are influenced by cultural behaviors [21] differences in dietary iron intake can also contribute to the observed variability of iron deficiency by country.

Similar to a previous population-based study conducted in the United States (3067 adults aged 70 and older, reporting 7% of women and 4% of men with iron deficiency based on the laboratory tests erythrocyte protoporphyrin, transferrin saturation, and serum ferritin) [5], we found higher incidence and prevalence of iron deficiency in women as compared to men. In younger populations, women have higher risk for iron deficiency than men due to physiological loss with the menstrual period [40, 41]. However, menstrual blood loss cannot explain those differences, since all participants were older than 70 years. This difference might be attributed to a lower dietary intake of iron in women [42], particularly meat [43, 44]. However, evidence on further explanations for sex-related differences in iron deficiency among older adults is still limited.

Interestingly, our findings suggest that age may influence the prevalence and incidence of iron deficiency in a relatively healthy population. In fact, a peak of iron deficiency is described in older adults, which is probably due to multifactorial reasons: poor diet, risk factors for inadequate iron absorption (for example medications, such as aspirin), or unphysiological iron loss (for instance through gastrointestinal diseases and oral anticoagulants) [9]. Similar to our findings, the EMPIRE study among 6267 adults in Portugal revealed a statistically significant higher prevalence rate of iron deficiency in participants aged ≥ 80 years compared to those aged < 65 and 65–70 years [36].

Based on the literature, among the different definitions of iron deficiency, low serum ferritin is supported as highly specific for iron deficiency when compared to the gold standard of stainable bone marrow iron [45]. On the other hand, since ferritin is an acute phase protein, its levels can be elevated in inflammatory states, chronic diseases, and malignancies, meaning that a normal ferritin may not always exclude iron deficiency [46]. That is why the definition based on ferritin alone might underestimate the prevalence of iron deficiency. Therefore, it is important that clinicians take into account an additional measurement, such as the sTfR and the sTfR-index. The fact that the large majority of our iron deficient study population did not have anemia highlights the need for iron assessment independent of hemoglobin levels. This further investigation of iron status is important, first because iron deficient people without anemia can suffer from a variety of physical symptoms in the same way or even worse as anemic people do [47], and second there is evidence that non-anemic iron deficiency is associated with increased overall mortality [17].

Our study has several strengths. First, we used four different definitions of iron deficiency. This spectrum makes the study results comparable to other studies at the measurement level, which may help to find a consensus on how to define iron deficiency among older adults in the future. Second, all blood samples were analyzed centrally in the same laboratory, contributing to a high reliability. Third, to the best of our knowledge, our study contributes unique data on iron deficiency among relatively healthy community-dwelling older adults from five Europe countries, both at the prevalence and the incidence level, over 3 years, and among 2157 participants.

There are also some limitations. The outcomes were measured on a yearly basis for only three consecutive years. Our results cannot be considered population-based as participants have been pre-selected to be relatively healthy older adults. However, this introduces a conservative bias on the prevalence and incidence of iron deficiency observed in our study.

## Conclusions

Our study shows that iron deficiency is frequent in relatively healthy European community-dwelling adults age 70 and older. Most vulnerable to iron deficiency in this target population were women, adults aged 80 + , and older adults from Portugal and Austria. Our findings at the methodological level comparing four measures of iron deficiency also suggest that ferritin alone may underestimate the prevalence of iron deficiency among older adults. Therefore, clinicians may be warranted to base their diagnosis of iron deficiency also on sTfR.

## Supplementary Information

Below is the link to the electronic supplementary material.

Supplementary file1 (DOCX 18 KB)
